# The prevalence of germline *DICER1* pathogenic variation in cancer populations

**DOI:** 10.1002/mgg3.555

**Published:** 2019-01-22

**Authors:** Jung Kim, Kris Ann P. Schultz, Dana Ashley Hill, Douglas R. Stewart

**Affiliations:** ^1^ Clinical Genetics Branch, Division of Cancer Epidemiology and Genetics National Cancer Institute, NIH Rockville Maryland; ^2^ Cancer and Blood Disorders Children's Minnesota Minneapolis Minnesota; ^3^ International Pleuropulmonary Blastoma/*DICER1* Registry Minneapolis Minnesota; ^4^ International Ovarian and Testicular Stromal Tumor Registry Minneapolis Minnesota; ^5^ Division of Pathology and Center for Cancer and Immunology Research Children’s National Health System Washington District of Columbia

**Keywords:** cancer population, *DICER1*, *DICER1* syndrome, prevalence estimate, TARGET, TCGA

## Abstract

**Background:**

The *DICER1* syndrome is an autosomal dominant tumor‐predisposition disorder associated with pleuropulmonary blastoma, a rare pediatric lung cancer. Somatic missense variation in “hotspot” codons in the RNaseIIIb domain (E1705, D1709, G1809, D1810, E1813) is observed in *DICER1*‐associated tumors. Previously, we found the prevalence of germline pathogenic *DICER1* variation in the general population is 1:10,600. In this study, we investigated the prevalence of pathogenic *DICER1* germline variation in The Cancer Genome Atlas (TCGA; 32 adult cancer types; 9,173 exomes) and the Therapeutically Applicable Research to Generate Effective Treatment (TARGET; two pediatric cancer types; 175 exomes) cohorts.

**Methods:**

All datasets were annotated and binned into four categories: pathogenic, likely pathogenic, variant of unknown significance, or likely benign.

**Results:**

The prevalence of *DICER1* pathogenic variants was 1:4,600 in TCGA. A single participant with a uterine corpus endometrial carcinoma harbored two pathogenic germline *DICER1* (hotspot and splice‐donor) variants, and a single participant with a rectal adenocarcinoma harbored a germline *DICER1* stop‐gained variant. In the smaller TARGET dataset, we observed no pathogenic germline variants.

**Conclusion:**

This is the largest comprehensive analysis of *DICER1* pathogenic variation in adult and pediatric cancer populations using publicly available data. The observation of germline *DICER1* variation with uterine corpus endometrial carcinoma merits additional investigation.

## 1 INTRODUCTION

*DICER1* (OMIM 606241) is an RNaseIII endonuclease that is crucial for processing pre‐miRNA into active mature miRNA. The *DICER1* syndrome is an autosomal dominant cancer predisposition disorder that arises from pathogenic germline variation in *DICER1* and is associated with a variety of benign and malignant tumors, including pleuropulmonary blastoma (PPB), cystic nephroma (CN), Sertoli‐Leydig cell tumors (SLCT), multinodular goiter (MNG), thyroid cancer, rhabdomyosarcoma, and pineoblastoma (Doros et al., 2014; Hill et al., 2009). *DICER1‐*associated tumors harbor somatic variation at amino acid “hotspot” residues located in the RNaseIIIb metal binding domain (E1705, D1709, G1809, D1810, and E1813), where alteration disrupts enzymatic function (Anglesio et al., 2013; Heravi‐Moussavi et al., 2012; Pugh et al., 2014; Seki et al., 2014). An unusual variation on Knudson's two‐hit hypothesis is observed in *DICER1*‐associated tumors: typically, the germline copy is a loss‐of‐function variant and the wild‐type copy is disrupted by a somatic hotspot variant that confers some retained function (Brenneman et al., 2015).

In previous work, we developed a scheme to classify germline *DICER1* variation modelled after the joint consensus recommendations of the American College of Medical Genetics and Genomics and the Association for Molecular Pathology (Kim, Field, Schultz, Hill, & Stewart, 2017). We applied this approach to germline *DICER1* variation from a variety of publicly available exome databases. In the largest (the portion of the Exome Aggregation Consortium excluding The Cancer Genome Atlas samples; *n* = 53, 103 exomes), we found the prevalence of *DICER1* pathogenic variation to be ~1/10,600, which was more common than expected (Kim et al., 2017). To better understand the neoplasia phenotype associated with *DICER1* variation, we now quantify the frequency of *DICER1* variation in cancer populations. In this study, we apply our published pathogenicity classification and investigate the prevalence of pathogenic germline (and somatic, when available) *DICER1* variants in publicly available genome datasets from cancer cohorts.

## 2 MATERIALS AND METHODS

### 2.1 The Cancer Genome Atlas (TCGA) datasets

The Cancer Genome Atlas used comprehensive genomic analyses to characterize germline and somatic variants in 32 adult tumor types (total: 9,173; http://portal.gdc.cancer.gov). Germline *DICER1* variation was downloaded from the Genomic Data Commons (GDC) application programming interface (API). BAM slicing of every subject with a blood‐derived normal sample, excluding acute myeloid leukemia (to avoid possibility of somatic variant contamination; 9,173 exomes, 32 cancer types, accessed 6/21/17; Table S1) was performed. The analyzed somatic data were downloaded directly from GDC.

### 2.2 Therapeutically applicable research to generate effective treatments (TARGET) datasets

The TARGET study used comprehensive genomic analyses to characterize germline and somatic variants; data are available for two pediatric tumor types (total: 175). Germline *DICER1* variation was extracted from the GDC API. BAM slicing of every subject with a blood‐derived normal sample, excluding acute myeloid leukemia (175 exomes, two cancer types, accessed 12/19/17) was performed.

### 2.3 Cancer variation resource (CanVar) publicly available dataset

CanVar used comprehensive genomic analyses to characterize germline variants in familial early‐onset colorectal cancer patients (total: 1,006). Germline *DICER1* variation was queried on http://canvar.icr.ac.uk (accessed 2/8/17).

### 2.4 Germline variant calling

Germline variants were called jointly by each cancer type using three different germline callers (GATK UnifiedGenotyper, GATK HaplotypeCaller, and FreeBayes), then assembled using bcbio‐nextgen (https://github.com/chapmanb/bcbio-nextgen). Variants called with at least two callers were used in the analysis.

### 2.5 Variant annotation, filtering, and classification

All exonic and splice‐site region (<10 intronic base pairs from intron/exon boundary) variants from the canonical *DICER1* transcript (NM_177438.2), including missense, frameshift, nonsense, and synonymous variants, were included. Multi‐allelic, deep intronic, and UTR variants were excluded in this analysis to focus on the protein‐coding regions. SnpEff (Cingolani et al., 2012) was used to annotate variants, and ANNOVAR (Wang, Li, & Hakonarson, 2010) was used to predict pathogenicity in silico, obtain population allele frequencies from different databases, and obtain previously reported variants from ClinVar (2017‐01‐25 version). Annotation by ClinVar and the Human Gene Mutation Database (HGMD, Qiagen and Institute of Medical Genetics, Cardiff, Wales, UK; version Professional 2017.1) was used to identify previously recognized and interpreted variants.

We used our published scheme to classify variants into pathogenic (P), likely pathogenic (LP), variant of uncertain significance (VUS), and likely benign (LB) categories (Kim et al., 2017). In brief, variants that are loss‐of‐function (LOF; e.g., nonsense and frameshift), located in the canonical splice site (≤2 intronic basepairs from the intron/exon boundary), missense located in *DICER1* hotspots (e.g., E1705, D1709, G1809, D1810, E1813) or credibly reported as pathogenic in at least one publication are classified as P. Classification as LP required a nonsynonymous missense variant to be outside a *DICER1* hotspot locus and harbor a bioinformatics pathogenicity prediction of “Deleterious” by metaSVM, a REVEL (Ioannidis et al., 2016) score >0.75, or a CADD (Kircher et al., 2014) score >30 (top 0.01% of the predicted deleteriousness). In somatic data, we considered loss‐of‐function and hotspot variation to be pathogenic.

### 2.6 Pathology review

Digital slides from tumors in TCGA *DICER1* P/LP carriers were subjected to review by an expert pathologist in *DICER1*‐associated tumors (DAH). Hematoxylin‐ and eosin‐stained images were obtained from cbioportal.org Cancer Digital Slide Archive (accessed 9/22/17 from the cbioportal.org tissue resource).

### 2.7 Ethical compliance

This study was performed using publicly available, peer‐reviewed, published datasets. No additional human‐subjects were involved.

## 3 RESULTS

### 3.1 Classification of unique germline *DICER1* variants

We identified 219 (TCGA), 24 (TARGET), and 11 (CanVar) unique exonic and splice‐site region *DICER1* variants (Tables 1 and Table S2). Most of the variants were classified as VUS and LB. For P/LP variation, 12 unique missense variants, one hotspot missense, one stop‐gained, and one splice‐donor were found in 17 individuals in TCGA (one person carried two pathogenic variants, p.Asp1810Asn [hotspot] and c.4206+1G>C [splice]). In TARGET, there was one unique missense variant, and one splice‐donor variant found in two carriers; no P/LP *DICER1* variants were observed in CanVar.

**Table 1:** Overview of The Cancer Genome Atlas (TCGA) *DICER1* unique variants

	TCGA (9,173 exomes)		
Total unique variants	219		
P	1 hotspot 1 stop‐gained 1 splice‐donor		
LP	metaSVM 9 missense	CADD 5 missense	REVEL 1 missense
VUS	18 splice regions 1 in‐frame deletion 1 missense		
LB	metaSVM 84 syn 103 missense	CADD 84 syn 107 missense	REVEL 84 syn 111 missense

Note

LB, likely benign; LP, likely pathogenic; P, pathogenic; syn, synonymous; VUS, variant of unknown significance.

### 3.2 Prevalence of pathogenic germline *DICER1* variants: TCGA

Of 32 cancer types available through TCGA (*n* = 9,173), we found 10 types (breast invasive carcinoma, bladder urothelial carcinoma, cervical squamous cell carcinoma and endocervical adenocarcinoma, lymphoid neoplasm diffuse large b‐cell lymphoma, thyroid carcinoma, head and neck squamous cell carcinoma, lung adenocarcinoma, rectum adenocarcinoma, ovarian serous cystadenocarcinoma and uterine corpus endometrial carcinoma) in 18 subjects that harbored a total of 16 unique (19 total including two in one person) germline P/LP *DICER1* variants (Table 2).

**Table 2:** The Cancer Genome Atlas *DICER1* pathogenic and likely pathogenic variation and tumor type

Cancer type (TCGA abbreviation; *n*)	Gender	Age at diagnosis (years)	Race	*DICER1 *germline	*DICER1 *somatic	Frequency of variant in database			Vital status
						ExAC non‐TCGA	ESP	1,000 genomes	
Breast invasive carcinoma (BRCA; 966)	Female	43	White	p.Gly1824Val	None	None	7.7 × 10^−5^	None	Living/disease free
Bladder urothelial carcinoma (BLCA; 394)	Female	82	Asian	p.Ser1160Tyr	None	4.71 × 10^−5^	None	None	Living/disease free Brother colon cancer
	Male	70	White	p.Ala1578Thr	None	None	None	None	Living/recurred/progressed Prior cancer history/mother breast cancer
Cervical squamous cell carcinoma and endocervical adenocarcinoma (CESC; 300)	Female	62	White	p.Leu1469Pro	None	1.89 × 10^−5^	None	3.99x10^−4^	Living/disease free
	Female	72	Asian	p.Ser1160Tyr	None	4.71 × 10^−5^	None	None	Living/disease free
Lymphoid neoplasm diffuse large B‐cell lymphoma (DLBC; 44)	Male	36	White	p.Ile528Thr	None	1.88 × 10^−5^	2.0 × 10^−4^	None	Living/disease free
Thyroid carcinoma (THCA; 431)	Female	64	N/A	p.Pro1836Leu	None	None	None	None	Living/disease free
**Rectum adenocarcinoma (READ; 153)**	**Female**	**62**	**N/A**	p.Glu904*	**None**	**None**	**None**	**None**	**Living/disease free**
Head and neck squamous cell carcinoma (HNSC; 509)	Male	61	White	p.Tyr1835Ser	None	None	None	None	Living/disease free
	Male	48	AA	p.Ile528Thr	None	1.88 × 10^−5^	2.0 × 10^−4^	None	Living/disease free
	Female	80	White	p.Arg1342His	None	None	None	None	Deceased
Lung adenocarcinoma (LUAD; 441)	Female	60	White	p.Phe1650Cys	None	None	None	None	Living/recurred/progressed
	Male	67	White	p.Trp1481Arg	None	None	None	None	Living/disease free
	Male	70	White	p.Arg201His	None	None	None	None	Deceased
Ovarian serous cystadenocarcinoma (OV; 415)	Female	39	White	p.Asp1390His	None	None	None	None	Deceased/recurred/progressed
Uterine corpus endometrial carcinoma (UCEC; 524)	**Female**	**57**	**AA**	**p.Asp1810Asn** **c.4206+1G>C**	**None**	**None**	**None**	**None**	**Living/disease free**
	Female	60	White	p.Trp1397Arg	None	None	None	None	Living/disease free
	Female	69	White	p.Ala1578Thr	*Asp1709Asn*, Arg937His	None	None	None	Living/disease free Prior cancer history

Note

**Bold text** denote Pathogenic germline variants, *italicized text* denote hotspot somatic variation.

AA, African American; ESP, exome sequencing project; ExAC non‐TCGA, portion of the exome aggregation consortium excluding samples from The Cancer Genome Atlas; N/A, not available.

Of the 16 P/LP germline *DICER1* variants in the TCGA dataset, 13 were unique missense alterations classified as LP, where in silico methods predicted a deleterious effect. Considering the in silico prediction tools separately, there were nine variants with a metaSVM score of “Deleterious,” five variants with a CADD score >30, and one variant with a REVEL score of >0.75. Of these 16 LP *DICER1*‐carriers, we found one patient with a uterine corpus endometrial carcinoma who harbored a p.Ala1578Thr germline *DICER1* variant and a somatic *DICER1* hotspot variant (p.Asp1709Asn). In addition, another subject with a uterine corpus endometrial carcinoma harbored two germline P *DICER1* variants: a splice‐donor c.4206+1G>C and an RNase IIIb missense (hotspot) p.Asp1810Asn. Thus in TCGA, the prevalence of *DICER1* P/LP was 1:700, 1:1,500, and 1:3,100 by metaSVM, CADD, and REVEL, respectively. Using a more stringent calculation by only considering P variants, the prevalence of *DICER1* P was ~1:4,600 (one subject with rectum adenocarcinoma and one subject with uterine corpus endometrial carcinoma; Table 3).

**Table 3:** Allele count (AC) of The Cancer Genome Atlas (TCGA) pathogenic (P) and likely pathogenic (LP) *DICER1* variation

	TCGA 9,173 subjects	AC
	# of unique variants	
Likely pathogenic	9 missense	11
Pathogenic	1 canonical splice variant, 1 hotspot	1
	1 stop‐gained	1
Pathogenic total AC		2
P/LP prevalence	1:706	
LOF (P) prevalence	1:4,600	

Note

LOF, loss‐of‐function.

### 3.3 Prevalence of pathogenic germline *DICER1* variants: TARGET

Of two cancer types (neuroblastoma [142 subjects] and Wilms tumor [33 subjects]) available through TARGET, we found only two subjects harboring LP *DICER1* variation, one with neuroblastoma and one with Wilms tumor (Tables S2 and S3), with a metaSVM score of “Deleterious” for two variants and CADD score >30 for one variant. Thus, in the TARGET cohort, the prevalence of *DICER1* P/LP was 1:88 by metaSVM and 1:175 CADD; using REVEL, no LP variants were found.

## 4 DISCUSSION

In this study, for the first time, we have comprehensively quantified the prevalence of P/LP germline *DICER1* variation in the largest publicly available sporadic adult and pediatric cancer cohorts. We observed an approximately twofold higher germline prevalence of the most damaging (pathogenic: LOF, splice, hotspot) of such variation in TCGA (9,173 subjects: 1:4,600) than was observed in non‐TCGA ExAC (53,103 subjects; 1:10,600) (Kim et al., 2017). In TCGA, we observed germline *DICER1* LOF, splice and hotspot variation in individuals with uterine and rectal cancers, which are not known to be germline *DICER1*‐associated. Such variation was not observed, however, in CanVar, a cohort of 1,006 early‐onset colorectal cancers. Below we comment on the germline *DICER1* (and when possible, somatic) variation observed in the adult (TCGA) and pediatric (TARGET) datasets.

Of the 32 types of tumors sequenced in the TCGA project with available *DICER1* germline data (Table S1), only one (thyroid carcinoma) has genetic and epidemiologic evidence of an association with pathogenic germline *DICER1* variation (Khan et al., 2017; Rutter et al., 2016). However, in the TCGA data, we did not observe any pathogenic variation in *DICER1* in the germline sequence for thyroid carcinoma; we did observe a LP (nonhotspot missense) variant in one participant with a thyroid carcinoma. One previous report found two TCGA thyroid cancers that harbored *DICER1* somatic hotspot variation (Wasserman et al., 2018). The lack of observed germline pathogenic *DICER1* variation in TCGA thyroid carcinomas may be secondary to study tissue requirements, which mandated sufficient tumor size with at least 60% tumor nuclei (https://cancergenome.nih.gov/cancersselected/biospeccriteria); this may have biased the study to more severe or aggressive tumors. In addition, the lack of pathogenic germline variation in *DICER1* in the TCGA study thyroid carcinoma samples may be attributable to its focus on sporadic (and adult) rather than familial cancers.

Of the 32 TCGA tumors with germline *DICER1* variation we analyzed (Table S1), four (testicular, breast, and prostate cancers and melanoma) have been reported in cohorts with germline *DICER1* pathogenic variation (*DICER1*‐carriers). A case series of 14 nonseminomatous testicular germ‐cell tumors found one germline mutation (Heravi‐Moussavi et al., 2012); subsequent work has cast doubt on a true *DICER1* association with testicular cancers (Conlon et al., 2015). In an analysis of 209 *DICER1*‐carriers from the International PPB/*DICER1* Registry and NCI *DICER1* syndrome study, a nonsignificant excess of breast cancer, prostate cancer, and melanoma was observed compared with US cancer registry (SEER) data (Stewart et al., 2019). In the current analysis, we did not observe any germline pathogenic variation in these four tumors, although one woman with breast cancer harbored a p.Gly1824Val nonhotspot missense (LP) variant. Taken together, it is unlikely that germline *DICER1* LOF, splice‐site, or hotspot variants contribute significantly to the risk of development of these tumors, bearing in mind the caveats of the TCGA study, noted above. The risk conferred by *DICER1* nonhotspot missense variation needs additional study.

There is one report of an association of colorectal cancer risk with 3′‐UTR polymorphisms in *DICER1* and other miRNA genes (Cho et al., 2015). In TCGA data, we observed germline pathogenic *DICER1* variation in one rectal adenocarcinoma. The rectum adenocarcinoma occurred in a 62‐year‐old female with a truncating *DICER1* variant; we did not observe any somatic P/LP *DICER1* variation in the associated tumor. We observed no germline P/LP *DICER1* variation in the 410 TCGA participants with colon adenocarcinoma or in the 1,006 individuals with familial early‐onset colorectal cancer from the CanVar study. Our analysis suggests that germline *DICER1* P/LP variation does not contribute significantly to the risk of development of colorectal cancers.

In one study of 290 endometrial tumors (Chen et al., 2015), six (2%) harbored *DICER1* somatic hotspot variation. In the 524 uterine corpus endometrial carcinomas in the TCGA study, one 57‐year‐old woman harbored both a heterozygous germline hotspot missense variant (p.Asp1810Asn) and a heterozygous germline canonical splice‐site variant (c.4206+1G>C); her uterine cancer contained these two germline variants but lacked an additional somatic *DICER1* variant. Given that *DICER1* is essential for embryogenesis, we hypothesize that these two variants are in *cis* rather than in* trans*. To date, *DICER1* hotspot variation has been observed in individuals mosaic for such variation, and not constitutionally (Brenneman et al., 2015; Klein et al., 2014). From the available TCGA data, it is not clear if the p.Asp1810Asn variant is constitutional or mosaic. Another possibility is age‐related somatic variation, commonly observed in *TP53* as clonal hematopoiesis (Genovese et al., 2014). Two women in the TCGA study with a uterine cancer each harbored a germline LP (nonhotspot missense) variant (p.Trp1397Arg; p.Ala1578Thr). In the woman with a germline p.Ala1578Thr variant, her tumor also harbored a known somatic hotspot variant (p.Asp1709Asn). Our review of the digital pathology available from the uterine corpus endometrial carcinomas from these patients with pathogenic germline variation showed no unusual morphologic features. In summary, 0.6% (3/524) of women in TCGA with a uterine corpus endometrial carcinoma harbored germline P/LP *DICER1* variation.

Biallelic *DICER1* variation is already known to account for a small percentage of Wilms tumors (Rakheja et al., 2014; Wu et al., 2013). The association of neuroblastoma and *DICER1* variation is unsettled. In the modest‐sized TARGET (pediatric) germline data available, we observed nonhotspot missense (LP) *DICER1* variation in one child with a neuroblastoma (one child with Wilms tumor also harbored a *DICER1* Likely Pathogenic variant). In an analysis of 209 *DICER1*‐carriers from the International PPB/*DICER1* Registry and NCI *DICER1* syndrome study, one case of neuroblastoma was observed, a nonsignificant excess compared with US cancer registry (SEER) data (Stewart et al., 2019). In two large (*n* = 240; *n* = 71) somatic sequencing studies of neuroblastoma, no somatic *DICER1* variation was observed (Pugh et al., 2013; Sausen et al., 2013). The risk conferred by constitutional bioinformatically predicted severe (LP) nonhotspot missense variation, akin to what we observed in one subject, remains uncertain. Similarly, the frequency of neuroblastoma arising from mosaic *DICER1* hotspot missense variation is unknown.

Limitations of this investigation include an inability to detect copy‐number changes in *DICER1* in the publicly available data. As noted above, the tissue requirements for TCGA mandated tumors with at least 60% tumor nuclei. The TCGA study also focused on sporadic rather than familial cancers. Survival biases may have influenced participant type in both TCGA and TARGET.

We report a range of *DICER1* pathogenic variant prevalence in adult and pediatric cancer populations drawn from large publicly available datasets. Compared with the general population prevalence (~1:10,600), the adult cancer cohort (TCGA: ~1:4,600) has trends toward greater frequency of pathogenic *DICER1* variation. Our observation of germline P/LP *DICER1* variation in 0.6% (3/524) of TCGA uterine corpus endometrial carcinoma merits additional investigation.

The content of this publication does not necessarily reflect the views or policies of the Department of Health and Human Services, nor does mention of trade names, commercial products, or organizations imply endorsement by the U.S. Government.

## CONFLICT OF INTEREST

The authors declare no relevant conflicts of interest.
