# Supplementary material for: The prevalence of germline DICER1 pathogenic variation in cancer populations
Source: Mol Genet Genomic Med. 2019 Jan 22;7(3):e555. doi: 10.1002/mgg3.555 (PMC6418698; doi:10.1002/mgg3.555)
Supplement: Supplementary file 1 [file MGG3-7-na-s001.docx]

Supplementary Table S1: TCGA cancer types and available cases for germline analysis

| Cancer Types | # of germline cases |
| --- | --- |
| Adrenocortical carcinoma | 87 |
| Bladder urothelial carcinoma | 394 |
| Brain lower grade glioma | 513 |
| Breast invasive carcinoma | 966 |
| Cervical squamous cell carcinoma and endocervical adenocarcinoma | 300 |
| Cholangiocarcinoma | 44 |
| Colon adenocarcinoma | 410 |
| Esophageal carcinoma | 126 |
| Glioblastoma multiforme | 485 |
| Head and Neck squamous cell carcinoma | 509 |
| Kidney chromophobe | 9 |
| Kidney renal clear cell carcinoma | 102 |
| Kidney renal papillary cell carcinoma | 232 |
| Liver hepatocellular carcinoma | 325 |
| Lung adenocarcinoma | 441 |
| Lung squamous cell carcinoma | 316 |
| Lymphoid neoplasm diffuse large B-cell lymphoma | 44 |
| Mesothelioma | 82 |
| Ovarian serous cystadenocarcinoma | 415 |
| Pancreatic adenocarcinoma | 153 |
| Pheochromocytoma and paraganglioma | 177 |
| Prostate adenocarcinoma | 440 |
| Rectum adenocarcinoma | 153 |
| Sarcoma | 237 |
| Skin cutaneous melanoma | 469 |
| Stomach adenocarcinoma | 396 |
| Testicular germ cell tumors | 150 |
| Thymoma | 112 |
| Thyroid carcinoma | 431 |
| Uterine carcinosarcoma | 50 |
| Uterine corpus endometrial carcinoma | 524 |
| Uveal melanoma | 80 |

Supplementary Table S2: Overview of Therapeutically Applicable Research to Generate Effective Treatments (TARGET) and CanVar *DICER1* variation. Abbreviations: P-pathogenic, LP-likely pathogenic, VUS-variant of unknown significance, LB-likely benign

|  | TARGET | | | CanVar |
| --- | --- | --- | --- | --- |
| Total Variants | 24 | | | 11 |
| LP | metaSVM  2 missense | CADD  1 missense | REVEL  0 missense | - |
| VUS | 2 splice regions  1 in-frame deletion | | | 4 splice regions |
| LB | metaSVM  9 syn  9 missense | CADD  9 syn  10 missense | REVEL  9 syn  11 missense | metSVM/CADD/REVEL  5 syn  2 missense |

Supplementary Table S3: Therapeutically Applicable Research to Generate Effective Treatments (TARGET) *DICER1* pathogenic and likely pathogenic variation and tumor type. Highlights denote pathogenic variants. ExAC-nonTCGA, Exome Sequencing Project and 1000 Genomes studies did not harbor any *DICER1* variation. *DICER1* somatic variation was not found in these patients. Abbreviation: ESP-Exome Sequencing Project

Pediatric Cancer (TARGET)

| Cancer Type | Gender | Age at Diagnosis | Race | *DICER1*  Germline | Vital Status |
| --- | --- | --- | --- | --- | --- |
| Neuroblastoma (142) | Male | 3 years | White | p.Ser1826Pro | Progressed/Deceased |
| Wilms Tumor (33) | Female | 5 years | White | p.Asp1502Asn | Living |
